# Morphofunctional State and Circadian Rhythms of the Liver under the Influence of Chronic Alcohol Intoxication and Constant Lighting

**DOI:** 10.3390/ijms222313007

**Published:** 2021-11-30

**Authors:** Maria A. Kozlova, Yuri A. Kirillov, Lyudmila A. Makartseva, Igor Chernov, David A. Areshidze

**Affiliations:** 1 Laboratory of Cell Pathology, A.P. Avtsyn Research Institute of Human Morphology, 117218 Moscow, Russia; ma.kozlova2021@outlook.com (M.A.K.); youri_kirillov@mail.ru (Y.A.K.); la.makartseva@outlook.com (L.A.M.); 2 Department of Pathological Anatomy, Tyumen State Medical University, 625023 Tyumen, Russia; chernov@tyumsmu.ru; 3 Experimental Tumor Chemotherapy Group, Center for Screening and Preclinical Testing, Institute of Problems of Chemical Physics of the Russian Academy of Science, 142432 Chernogolovka, Russia

**Keywords:** alcohol, liver, hepatocyte, circadian rhythm

## Abstract

A study of the influence of chronic alcohol intoxication, constant illumination and their combined effects on the morphofunctional state of the rat liver and the circadian rhythms (CR) of the studied parameters of the organism was carried out. It was found that both alcohol and constant illumination caused significant changes in the structure of the liver, as well as in the circadian rhythmicity of micromorphometric parameters of hepatocytes, ALT, and total and direct bilirubin rhythms; however, the combined effects of ethanol and constant illumination had the most significant effect on the studied parameters of the organism. These two factors caused disturbances in the circadian rhythms of the micromorphometric parameters of hepatocytes, disruption of the circadian rhythms of total protein, albumin, AST, ALT, and direct and total bilirubin, as well as disturbances in the expression and rhythmicity of the studied clock genes against a background of the development of an inflammatory process in the liver.

## 1. Introduction

Alcohol abuse is one of the most important health and social problems in modern society. Furthermore, the liver is one of the organs most significantly affected by alcohol intoxication. Systematic alcohol consumption leads to disruption of the structure and function of the liver, which, in turn, alters the metabolism of other organs. Alcoholic liver disease (ALD) include several sequential clinical and morphological forms, which are stages of a single pathological process caused by prolonged alcohol use [1,2].

The rhythmicity of vital processes, which is one of the fundamental properties of living matter, includes periodic changes and the integration of various processes specific to biological systems at different levels of organization [3]. For biological processes, rhythms with different frequencies from fractions of a second to tens of years are described, but one of the most significant types of biorhythms in mammals, including humans, are diurnal or circadian rhythms (CR) [4,5]. The separate CRs of distinct biological processes in the various organ systems form a precisely coordinated ensemble—the chronostructure of the organism. The presence of an organized rhythmic structure of biological processes provides the necessary order of their occurrence, which determines the maintenance of the functioning of body systems at an optimal level [6,7,8,9].

The coordination of mammalian CRs is genetically determined, but at the same time, it is plastically modulated under the influence of periodic factors in the external and internal environment—synchronizers, or pacemakers [10,11]—the leading role of which is played by the light cycle. Violation of the normal rhythm (changes in the amplitude and/or phase characteristics of the rhythm) of the vital processes of an organism causes the appearance of desynchronosis, which can lead to the development of diseases and pathological conditions [12,13,14,15,16].

To date, it has been established that most of the CRs of the liver, as in other organs, are autonomous in the absence of the influence of external pacemakers. In hepatocytes, the biological clock at the molecular level, which ensures CR autonomy, includes the *Bmal* gene paired with the *Clock* gene, the *Per* gene family (*Per1, Per2, Per3*), and the *Cry* genes (*Cry1*, *Cry2*, encoding cryptochrome proteins) [17,18]. It has been shown that the rhythm of expression of clock genes persists in the dark, but also that a number of rhythms can be destroyed [19,20]. It has been proven that the main function of changes in light and darkness, as a pacemaker, is to influence the CR period and the amplitude, gene expression, and harmonization of rhythms with each other [21].

The main drivers of circadian rhythms in mammals are the suprachiasmatic nuclei of the hypothalamus (SCN). From the outside, the rhythm-organizing function of the SCN is modulated by environmental time setters, the main of which is light. In addition, correction of the functional state of pacemakers in the brain and beyond is carried out by various neurotransmitters and hormones. The leading role in the external regulation of liver CRs belongs to the hypothalamus–pituitary–adrenal axis and the pineal gland [22]. Another time setter that determines the CR structure of a number of organs, including the liver, is feeding. Metabolic processes easily get out of control of the SCN when food intake is desynchronized with normal daily activity. In this situation, mealtime (feeding time) becomes the dominant control factor [23,24,25].

A significant factor in the disorganization of biorhythms in the modern world is so-called light pollution, which causes a disruption of the light–dark cycle. For many reasons of social origin (overtime and shift work, prolonged interaction with digital equipment, transmeridian flights, etc.), a modern person is almost inevitably exposed to abundant exposure to artificial lighting at night, which often leads to the development of desynchronosis [26]. At present, the effect of light at night on CRs is well described. It has been established that light pollution, provoking desynchronosis, can be one of the causes of a number of pathologies [27]. It was shown that changes in lipid and carbohydrate metabolism correlate with the level of light pollution [28,29], disruptions of the light cycle are one of the possible preconditions for the occurrence of metabolic syndrome and can increase the risk of developing type 2 diabetes mellitus and atherosclerosis [16,30]. There is evidence that changes caused by chronic desynchronosis can lead to the development of malignant liver tumors [31,32].

Likewise, ethanol has a pronounced chronodestructive effect on the CRs of organisms that largely determines increases in the susceptibility of the gastrointestinal tract and liver to alcohol-induced damage and makes a significant contribution to the severity of alcohol pathology [33,34,35,36,37]. As such, in alcoholism, CR synchronization disorders are one of the first symptoms [38,39].

A large amount of data [35,40] has shown that *Clock*- and *Per2*-knockout mice develop alcohol-induced steatosis, fibrosis, and cirrhosis of the liver much faster than controls. In turn, inflammation and cytokines, being two of the key components of the pathogenesis of alcoholic liver damage [41,42], can also disrupt the normal functioning of circadian genes [43,44].

Nevertheless, the literature available to us does not describe the effects of the joint impact of chronic alcohol intoxication (CAI) and constant lighting both on the structure of the liver and on its CRs, although both of these factors are chrono-destructive and affect the morphofunctional integrity of this organ. Based on this, we conducted a study of the effect of constant illumination, 21-day alcohol intoxication and the combined influence of these factors on the structure, function and some CRs of the liver.

The modification of morphological state of hepatocytes, which has a wide range of variations, is a reflection of their functional changes [45]. The linear dimensions of hepatocytes and their nuclei, their nuclear–cytoplasmic ratio, a number of other micromorphometric parameters, as well as some biochemical and immunohistochemical parameters, are significant indicators for assessing the state of the liver [46].

## 2. Results

### 2.1. Influence of Constant Lighting and CAI on the Morphofunctional Condition of the Liver

**Figure 1:**
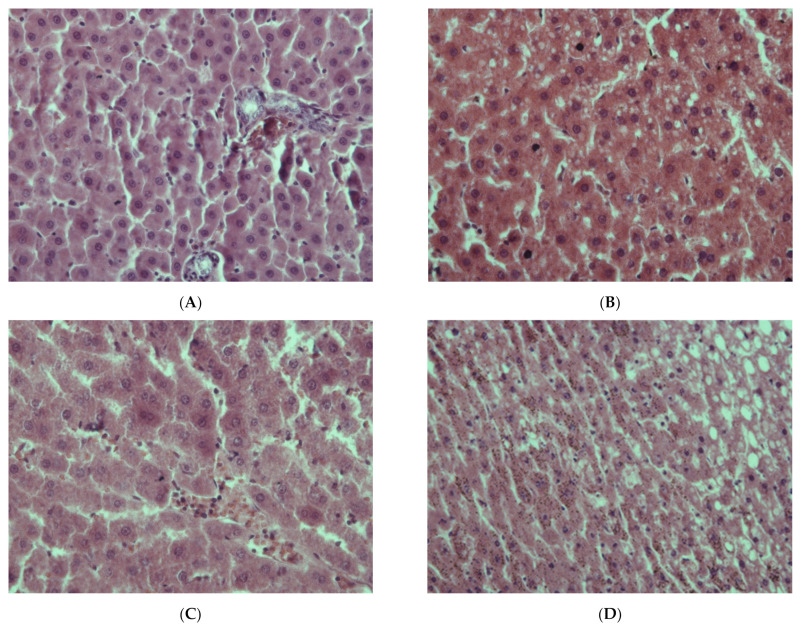

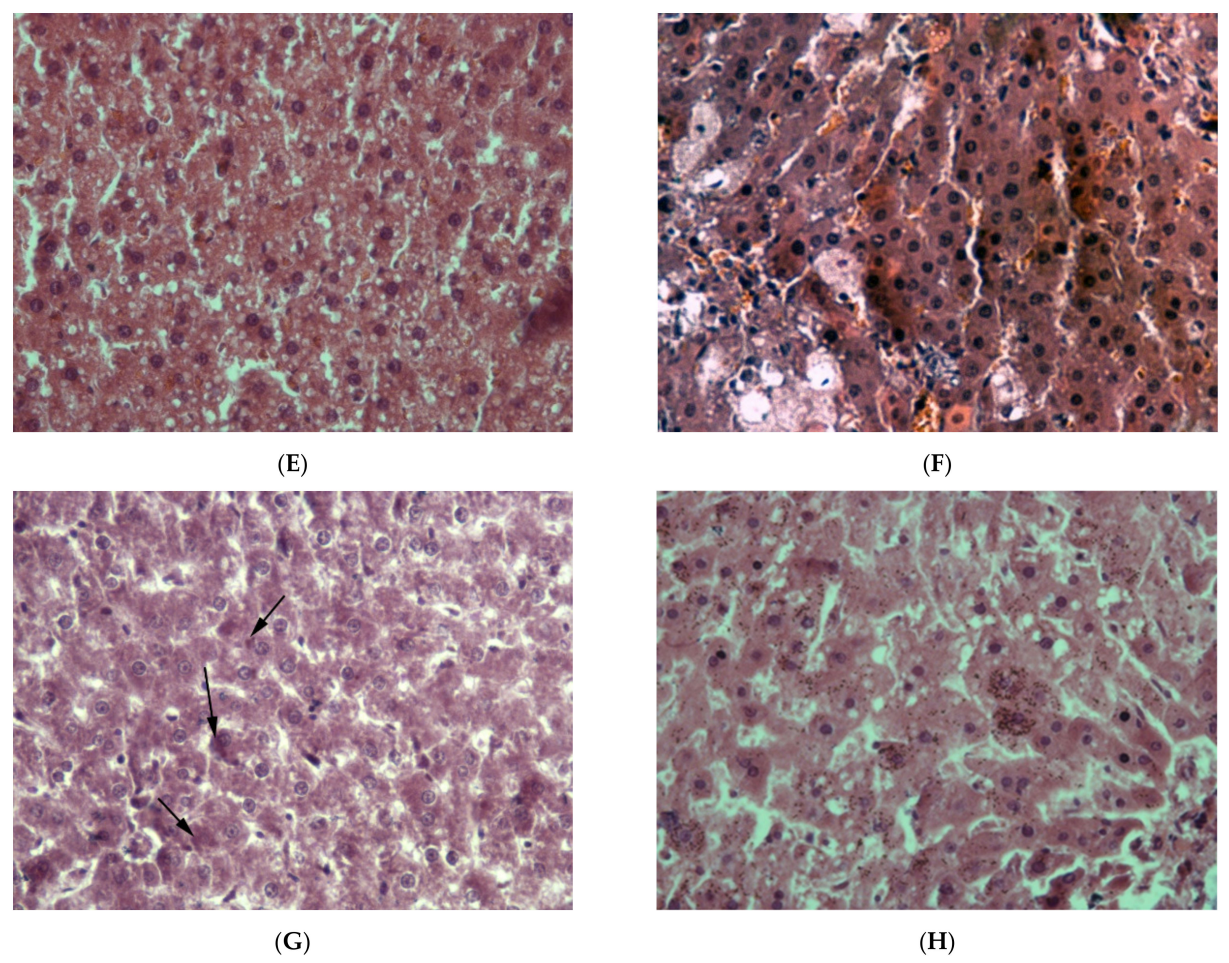
Liver of rats: (**A**)—Control; (**B**)—EtOH; (**C**)—CL; (**D**–**F**)—EtOH + CL; (**G**)—EtOH + CL, arrows indicate the Mallory–Denk bodies; (**H**)—EtOH + CL, Hematoxylin–eosin staining. (**A**–**C**,**E**–**H**) ×400, (**D**) ×200. Note: hereafter, Control—control group, EtOH—first experimental group, CL—second experimental group, EtOH + CL—third experimental group.

**Figure 2:**
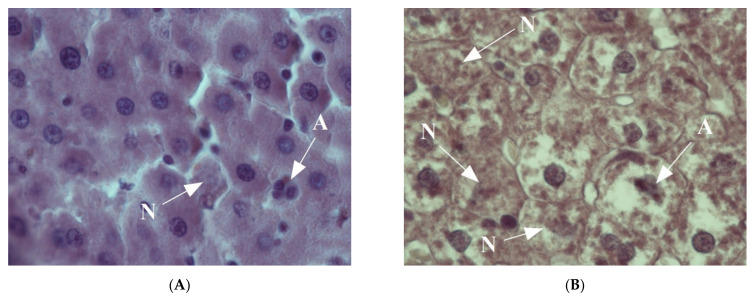
Livers of rats: (**A**)—Control; (**B**)—EtOH + CL. Arrows show the cells in conditions of: A—apoptosis; N—necrosis. Hematoxylin–eosin staining, ×1000.

The morphological pattern of the liver in the rats of the control group corresponded to the age norm, i.e., the liver had a preserved structure of hepatic cords, composed of polygonal hepatocytes with a rounded, centrally located nucleus without signs of dystrophic changes and necrosis (Figure 1A). In the liver of rats in the first experimental group, a significant number of vacuole-containing hepatocytes were found (Figure 1B). The control staining with Sudan-III verified the presence of lipid drops in the cytoplasm of the hepatocytes in this group, which indicates the beginning of the development of fatty degeneration of the liver. At the same time, in this group, we observe both single cells and groups of hepatocytes in a state of necrosis and apoptosis (Figure 2A).

The livers of rats of the second experimental group retained a practically normal structure, but single necrotic hepatocytes appeared in them, as well as cells with signs of fatty degeneration, in which the presence of small lipid-containing vacuoles was noted (Figure 1C).

**Figure 3:**
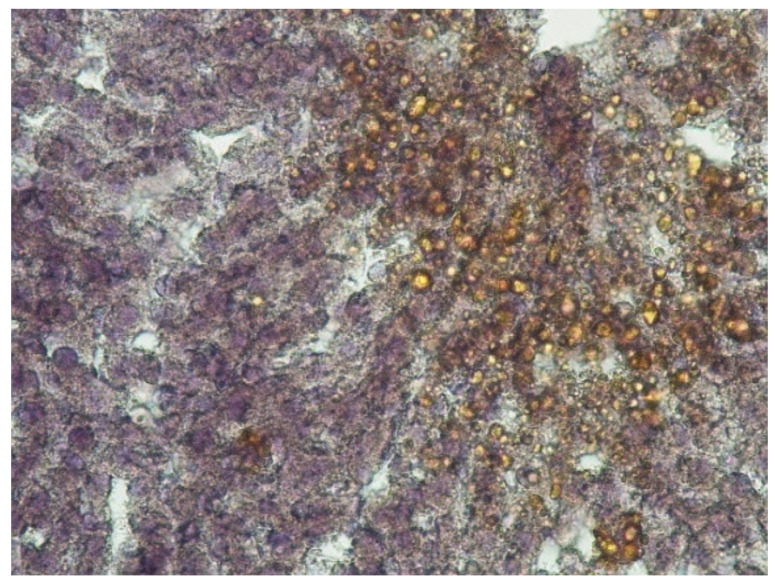
Lipid-containing vacuoles in the hepatocytes. EtOH + CL. Sudan-III staining with hematoxylin afterstain, ×200.

In the livers of rats of the third experimental group, a pattern representative of the development of alcoholic hepatitis was revealed (Figure 1D–F); inclusions of lipofuscin and Mallory–Denk bodies were also found (Figure 1G,H). Numerous necrotic cells were noted, as well as cells in the process of apoptosis (Figure 2B), and infiltrates consisting of neutrophilic leukocytes, lymphocytes and macrophages. A significant portion of the hepatocytes were in a state of small- and large-droplet fatty degeneration (Figure 3). The cord structure of the organ was violated, and the small monomorphic regenerate nodes (false lobules), separated by narrow layers of connective tissue, were revealed.

The results of the karyometry showed that the perimeters of the hepatocyte nuclei, as well as their cross-sectional areas and volumes in the first and second experimental groups largely corresponded to the parameters of the control group; however, in the third experimental group, there was a significant decrease in these parameters as compared to the controls, and the ratio of the nucleus volume to the area was also significantly reduced (Table 1)—at the same time, the NCR values in this group were significantly lower than in the first group, and were the lowest in the second group.

The value of the mean diameter of the nuclei of hepatocytes, on the contrary, was significantly higher in the second group in comparison to the control parameters— which also indicates a change in the elongation index and shape of the nuclei.

While constant illumination (second group) led to a significant increase in the cross-sectional area of hepatocytes, a significant number of small cells were detected in the liver under the combined effect of ethanol and constant illumination (third group), which was manifested by a decrease in the mean cross-sectional area of the hepatocytes in comparison to the second group, to values close to those of the control group. In the third group, changes were noted only in terms of the parameters of the shape of nuclei (contour index, coefficient of form), while the values of their diameters and elongation indices were identical to those of the control group. Changes in the proportion of binuclear cells were statistically significant only in the livers of animals in the second group.

### 2.2. Study of the Diurnal Dynamics of Cross-Sectional Areas of Nuclei, Areas of Cells, and NCR

**Figure 4:**
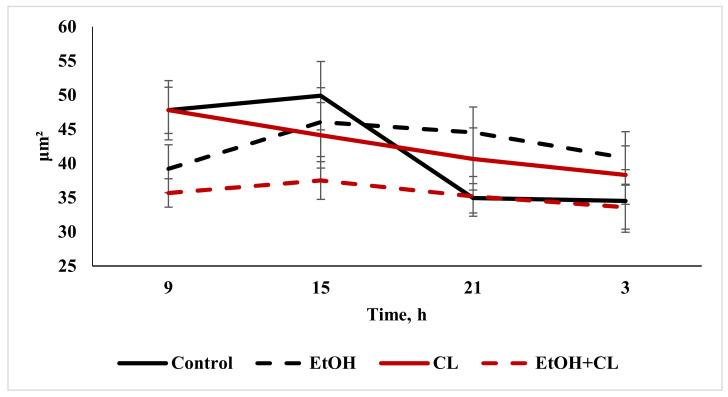
Daily dynamics of area of nuclei of hepatocytes of rats.

**Figure 5:**
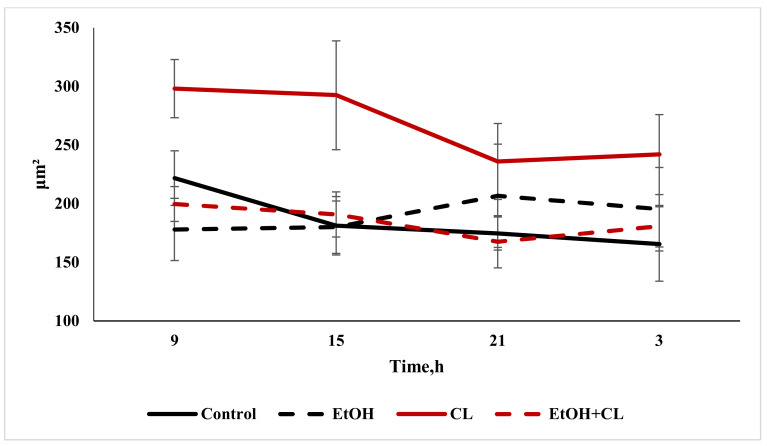
Daily dynamics of area of hepatocytes in rats.

**Figure 6:**
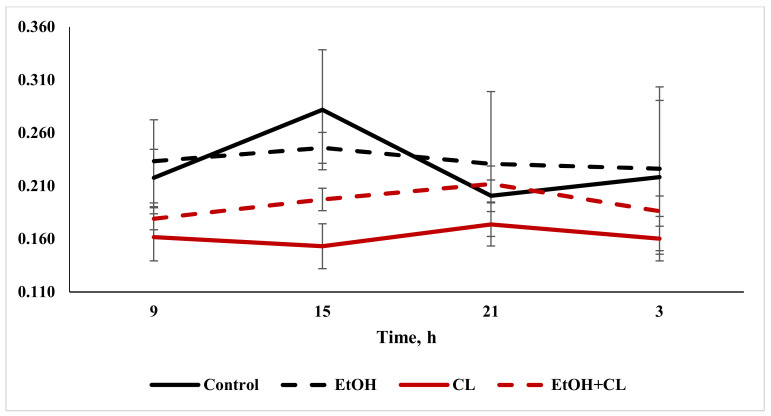
Daily dynamics of NCR in hepatocytes of rats.

When considering the daily dynamics of the studied parameters, it was found that they significantly differed in the experimental groups from the control parameters (Figure 4, Figure 5 and Figure 6).

The results of the cosinor analysis (Table 2) indicated the presence of a reliable circadian rhythm in the parameters of the area of the nuclei, the area of the cell and the NCR in the hepatocytes of the rats in the control group. In the first experimental group, as a result of three weeks of alcohol intoxication, the CRs of the cell area and the NCR were destroyed and the rhythm of the nuclear area was shifted (Table 2).

Constant lighting led to the destruction of the rhythm of the NCR and a significant shift in the rhythms of nuclei and cells (Table 2).

In the third experimental group, despite the disrupted rhythm of the nucleus area, the rhythms of the cell area and the NCR were preserved (Table 2).

### 2.3. Influence of Constant Lighting and CAI on Several Biochemical Parameters

**Figure 7:**
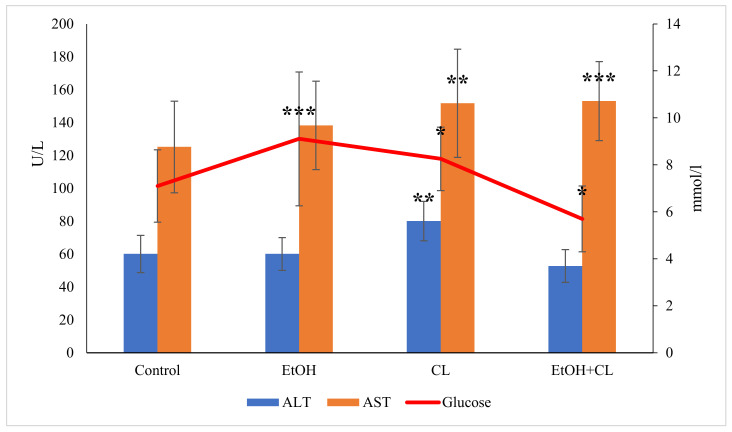
Glucose content and activity of ALT and AST in the blood plasma of rats, * *p* < 0.05, ** *p* < 0.01, *** *p* < 0.001.

Analysis of glucose content in the blood plasma of rats allowed us to establish that, in animals of the first and second groups, the value of this parameter increased from 7.10 ± 1.51 mmol/L in the control group to 9.11 ± 2.88 mmol/L and 8.26 ± 1.35 mmol/L, respectively—at the same time, in animals of the third group, the glucose content was much lower: 5.70 ± 1.40 (Figure 7; Table 3).

The value of ALT was almost unchanged in the first and third experimental groups, at 60.12 ± 9.93 U/L and 52.81 ± 10.00 U/L, respectively, against 60.16 ± 11.37 U/L in the control group. The activity of ALT in the blood plasma of rats in the second group increased to 80.10 ± 10.50 U/L, which was higher than the values of all the other groups (Figure 7).

The activity of AST in the control group was 125.2 ± 27.90 U/L; in the second and third groups it increased significantly to 151.80 ± 32.99 U/L and 153.1 ± 24.06 U/L, respectively.

**Figure 8:**
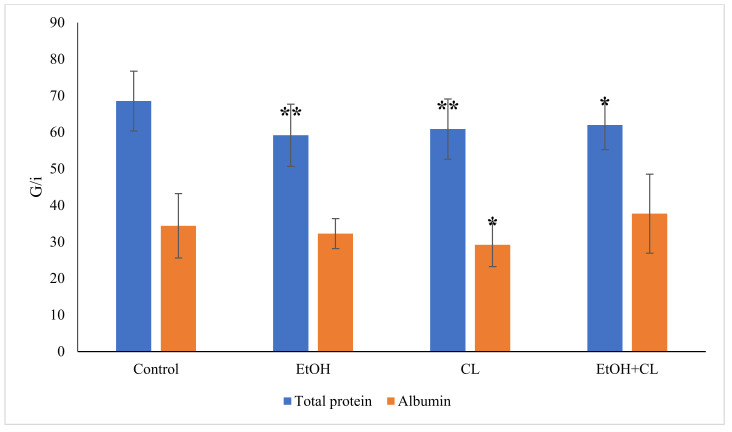
Total protein and albumin content in the blood plasma of rats, * *p* < 0.05, ** *p* < 0.01.

The total protein content in the blood plasma of animals in the control group was 68.55 ± 8.19 g/L; at the same time, in the blood plasma of rats in the experimental groups it was lower: 59.71 ± 8.52 g/L in the first, 60.87 ± 8.22 g/L in the second and 61.96 ± 6.68 g/L in the third group (Figure 8).

The albumin content decreased significantly only in the second group, at 29.17 ± 5.91 g/L against 34.44 ± 8.80 g/L in the control group (Figure 8).

**Figure 9:**
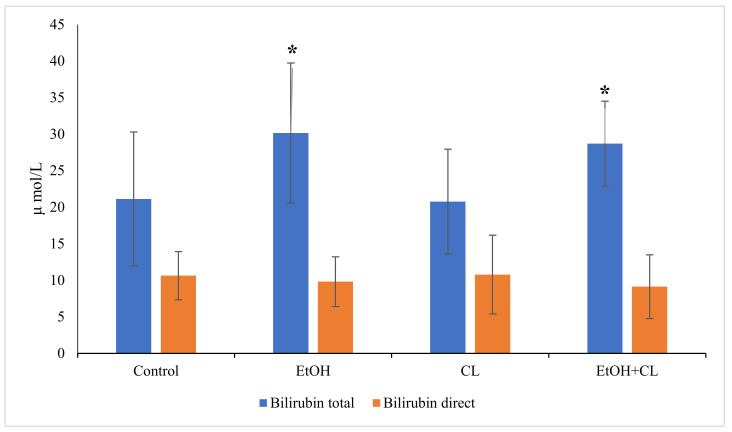
Content of direct and total bilirubin in the blood plasma of rats, * *p* < 0.05.

When analyzing the content of direct bilirubin, there were no significant differences from the control level of 10.64 ± 3.33 µmol/L. Total bilirubin content increased significantly in the blood of animals in the first and third groups, reaching levels of 30.15 ± 9.50 µmol/L against 21.12 ± 9.16 µmol/L in the control group (Figure 9).

### 2.4. Influence of Constant Lighting and CAI on the Organization of the Circadian Rhythms of Several Biochemical Parameters

**Figure 10:**
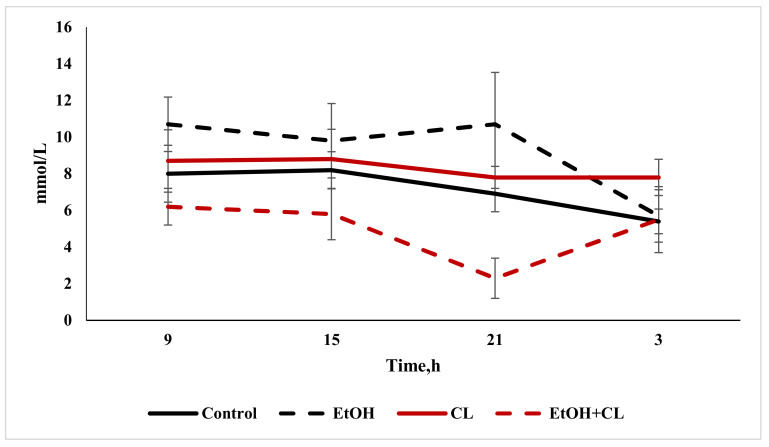
Daily fluctuations in blood glucose of rats.

Analysis of the daily rhythm of glucose allowed us to detect the presence of a maximum level of this parameter at 15:00, with minimum values at 3:00 in the blood plasma of rats in the control group. In animals of the first group, at a maximum at 21:00, the same minimum was maintained. In the second group, the same extrema as in the control group were highlighted on the smoothed chronogram, and in the animals of the third group, the maximum fell at 9:00, with a minimum at 21:00 (Figure 10).

The results of the cosinor analysis indicated the presence of reliable CRs for this parameter in animals of all groups. With sufficiently pronounced amplitude fluctuations, the acrophases of the rhythm occurred during the daytime in all groups (Table 3).

**Figure 11:**
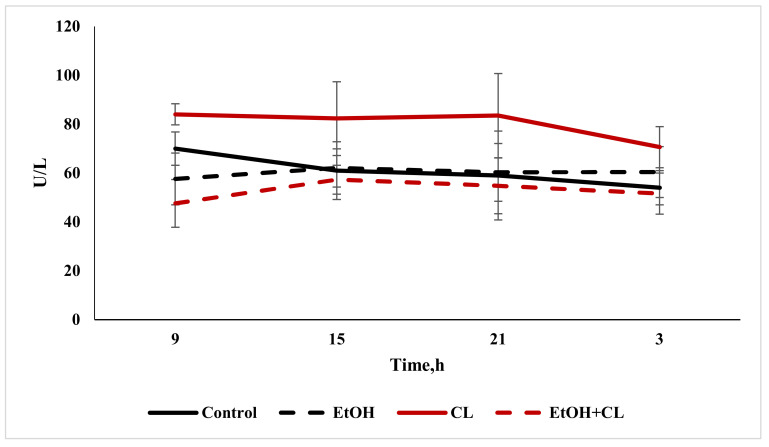
Daily fluctuations in the activity of ALT in the blood plasma of rats.

The diurnal rhythm of ALT in the blood plasma of rats in the control group showed its maximum at 9:00 and its minimum at 3:00. In animals of first group, the maximum value shifted at 15:00, with the minimum at 9:00. The ALT rhythm in the second group was a little more pronounced than in the control, with the same extrema. In the blood plasma of rats of the third group, the maximum activity of the enzyme was noted at 15:00, and it was least active at 9:00 (Figure 11).

The results of the cosinor analysis showed the presence of a reliable CR of activity of ALT in rats of the control group only.

**Figure 12:**
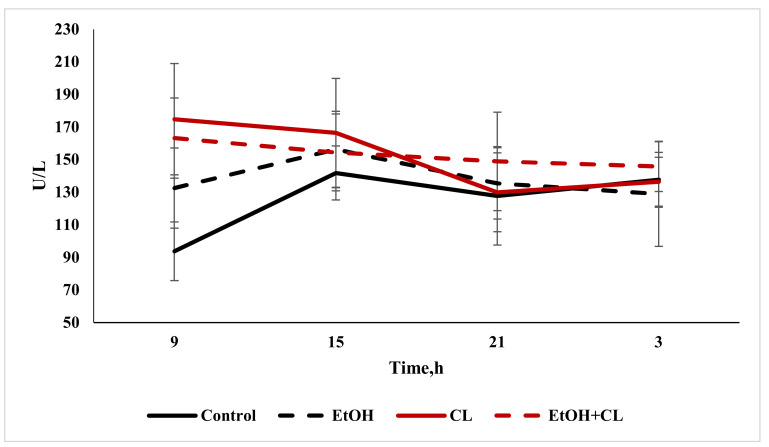
Daily fluctuations in the activity of AST in the blood plasma of rats.

Analysis of the daily activity of AST in the blood of rats in the control group made it possible to establish the presence of a maximum at 15:00 with a minimum at 9:00. In the first group, with the same maximum, the minimum point of the rhythm shifted at 3:00; in the second group, with a maximum at 9:00, the minimum values were noted at 21:00; and the chronogram of the third group was characterized by a maximum value at 9:00, and a minimum at 3:00 (Figure 12).

Cosinor analysis showed the presence of a reliable CR of activity for AST in the control group, and also in animals of the first and second control groups, which significantly differed from the control parameters.

**Figure 13:**
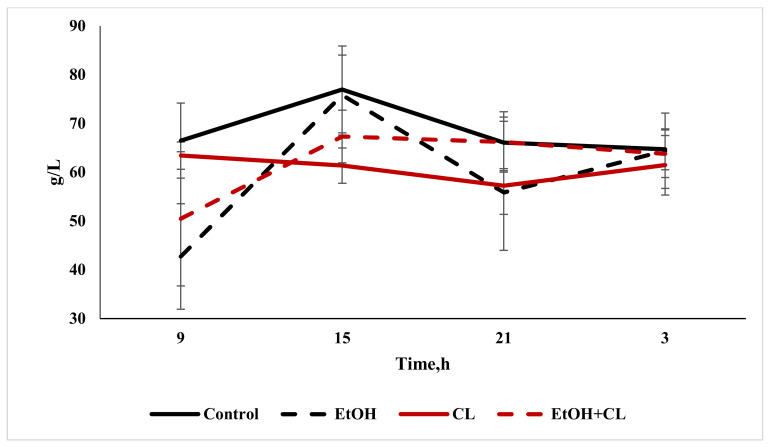
Daily fluctuations in total protein levels in the blood plasma of rats.

The maximum total protein content in the blood plasma of rats in the control group was found at 15:00, with a decrease at 3:00. Under the influence of ethanol under fixed lighting conditions, the maximum total protein content occurred at 15:00, with a minimum at 9 h; under constant lighting, the maximum total protein level was noted at 9:00, and the minimum at 21:00. In the blood plasma of rats in the third group, the lowest protein content was noted at 9:00, with the maximum occurring at 15:00 (Figure 13).

Cosinor analysis made it possible to establish the CRs of the total protein levels, differing in their phase-amplitude characteristics, in each group—except for the third.

**Figure 14:**
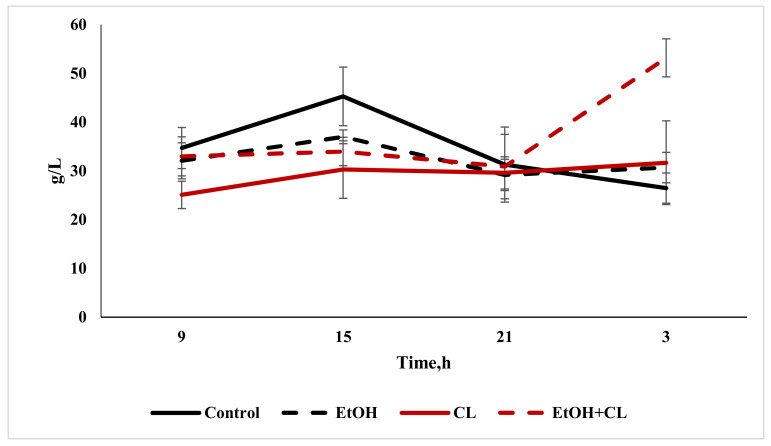
Daily fluctuations in albumin levels in the blood plasma of rats.

The maximum content of albumin in the control group was noted at 15:00; furthermore, its content decreased to a minimum at 3:00. In animals of the first group, with the same maximum, the minimum albumin content was found at 21:00—while in the second group, the minimum level was noted at 9 h, with a maximum at 3:00. In the third group, with a minimum at 21:00, the levels of this protein increased significantly by 3:00 (Figure 14).

As in the case of total protein levels, the same CRs for albumin were found in the control group and in the first and second experimental groups.

**Figure 15:**
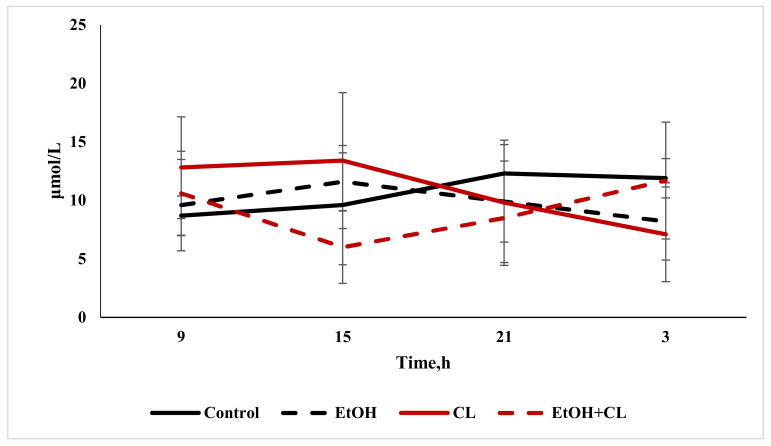
Daily fluctuations of direct bilirubin content in the blood plasma of rats.

The maximum value of direct bilirubin content in the blood of control rats was found at 21:00 and the minimum at 9:00. In the rats of the first group, with a maximum at 15:00, the decrease in bilirubin content to a minimum occurred at 3:00. In animals of the second group, the maximum value of direct bilirubin content was also noted at 15:00, with a minimum at 3:00—but in rats of the third group on the other hand, the maximum occurred at 3:00, and the minimum at 15:00 (Figure 15).

The results of the cosinor analysis made it possible to establish a reliable CR of direct bilirubin in rats of the control group only.

**Figure 16:**
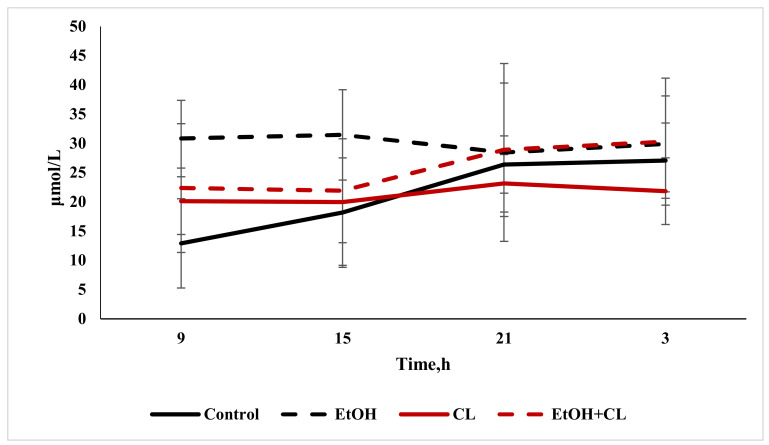
Daily fluctuations of total bilirubin in the blood plasma of rats.

Daily fluctuations in the level of total bilirubin in the blood plasma of rats in the control group were characterized by a minimum at 9:00 and maximum values at 3:00. In the first and second groups, the rhythm was significantly smoothed—in the former with a maximum at 15:00 and a minimum at 21:00; in the latter, contrarily, the maximum was noted at 21:00, with a minimum at 15:00. In the third group, maximum values of total bilirubin level were found at 3:00, and minimum values at 15:00 (Figure 16).

According to the results of the cosinor analysis, a reliable CR for this parameter was noted in the control group only.

### 2.5. Influence of Constant Illumination and CAI on Gene Expression

Results of immunohistochemical studies testify that the proportion of *Ki-67*-positive cells significantly increased in the liver of rats in the third group. The intensity of *p53* expression was significantly higher than in the controls in the hepatocytes of rats from all the experimental groups. At the same time, in animals of the experimental groups, the proportion of *Bmal1*-positive cells significantly decreased, but the number of cells displaying *Per2* expression increased. Expression of *Adh5* significantly increased in the liver cells of animals in the first and third experimental groups (Table 4).

### 2.6. Influence of Constant Lighting and CAI on the Organization of Circadian Rhythms for the Expression of Several Genes

**Figure 17:**
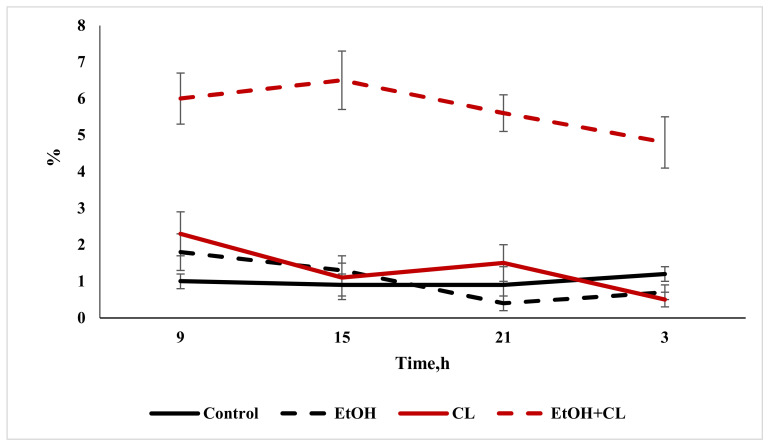
Daily fluctuations in the expression of *Ki-67* in rat hepatocytes.

When studying the diurnal dynamics of *Ki-67* expression, we found that in the control group, the maximum expression was observed at night, and the minimum in the daytime. In the hepatocytes of rats of the first experimental group, the maximum expression was noted at 9:00, and the minimum at 21:00. In the rats of the second group, showing the same maximum, the minimum values were noted at 3:00. In the hepatocytes of animals of the third group, the maximum expression of *Ki-67* was noted at 15:00, and the minimum at 3:00 (Figure 17).

The cosinor analysis made it possible to establish reliable CRs for *Ki-67* expression in the hepatocytes of rats of the control group and the first group, with pronounced amplitude-phase differences between them (Table 4).

**Figure 18:**
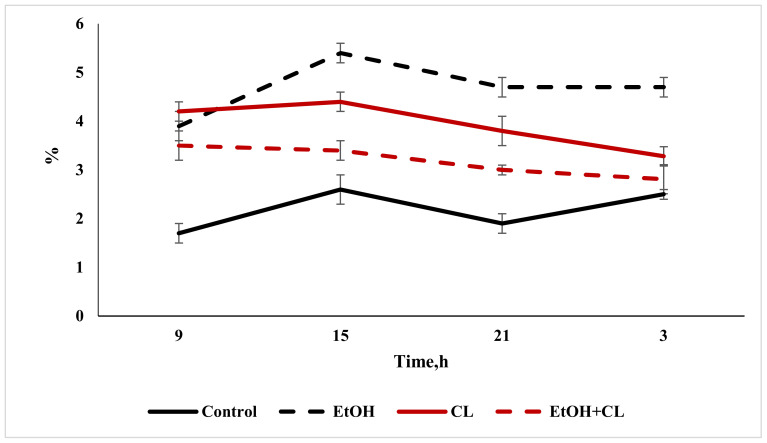
Daily fluctuations in the expression of *p53* in rat hepatocytes.

When considering the daily dynamics of *p53* expression in the control and first experimental group, the maximum gene expression was found at 15:00, with a minimum at 9:00. In animals of the second group, the minimum occurs by 3:00, and in rats of the third group, with the same minimum at 3:00, the maximum values were noted in 9:00. Chronograms in the last two groups are smoothed (Figure 18).

The cosinor analysis conducted showed the presence of significant CRs for *p53* expression in the liver of rats of the control and first groups (Table 5).

**Figure 19:**
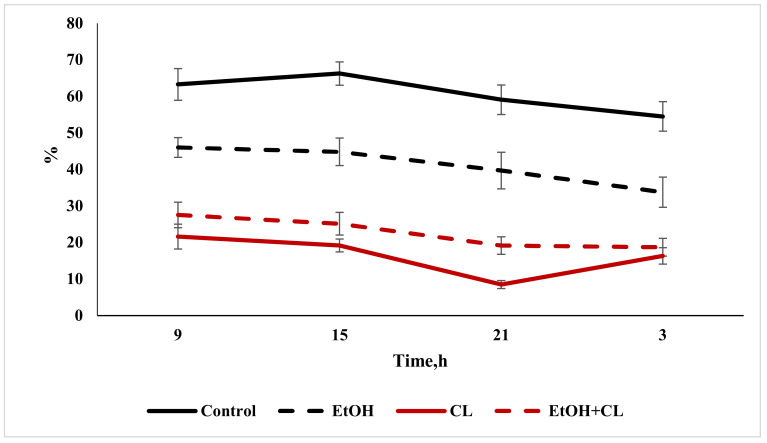
Daily fluctuations in the expression of *Bmal1* in rat hepatocytes.

When studying the daily dynamics of *Bmal1* expression in the hepatocytes of rats of the control group, we found a maximum at 15:00 and a minimum at 3:00. In animals of the first and third experimental groups, the maximum was noted at 9:00, with the same minimum as in the controls. In the hepatocytes of rats of the second group, with a maximum at 9:00, the minimum shifted by 21:00 (Figure 19).

At the same time, the results of the cosinor analysis showed the presence of reliable CRs for this parameter only in the hepatocytes of the animals of the control and the second experimental groups.

**Figure 20:**
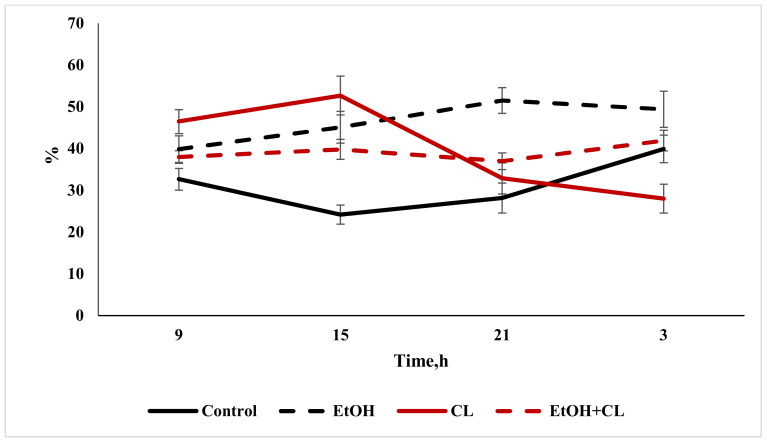
Daily fluctuations in the expression of *Per2* in rat hepatocytes.

The daily rhythmicity of *Per2* expression in the hepatocytes of intact animals was characterized by a maximum at 3:00 and a minimum at 15:00. In the liver of rats in the first experimental group, the maximum shifted by 21:00, and the minimum expression was noted at 9:00. In the animals of the second group, with a maximum at 15:00, the minimum was displaced by 3:00, and in the third group, with a smooth rhythm, the maximum was noted at 3:00 and the minimum at 21:00 (Figure 20).

The cosinor analysis also showed the presence of reliable CRs for the expression of *Per2* in the control and second experimental groups.

**Figure 21:**
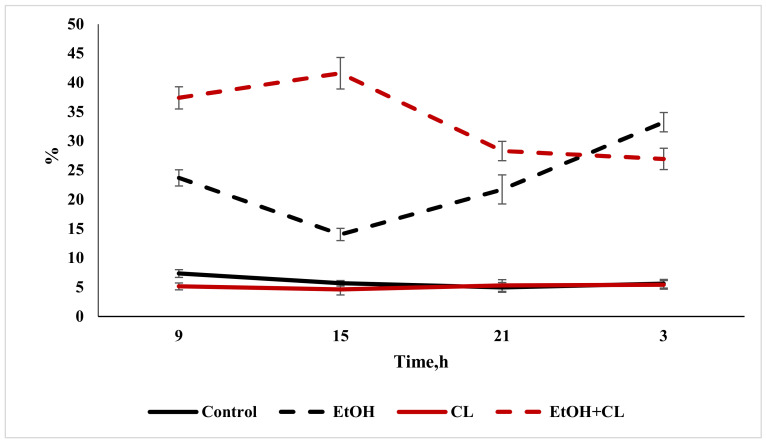
Daily fluctuations in the expression of *Adh5* in rat hepatocytes.

In the hepatocytes of rats in the control and second experimental groups, daily fluctuations of *Adh5* were extremely weak—the maximum expression in the controls was found at 9:00, with a minimum at 21:00; in the second group, both extrema were shifted to the previous time points. In the rats of the first experimental group, the maximum was noted at 3:00, and the minimum at 15:00; in the animals of the third group, the pattern of the rhythm was opposite (Figure 21).

According to the cosinor analysis, CRs for *Adh5* expression were observed in the control group, as well as in the hepatocytes of the animals of the first and third experimental groups.

## 3. Discussion

The analysis of pathomorphological changes in the liver allowed us to establish that the joint influence of two factors—constant lighting and chronic alcoholic intoxication—leads to significant alterations in the morphofunctional condition of the liver within three weeks.

In particular, if the influence of alcohol under a regime of alternating light and darkness provokes the occurrence of fatty degeneration of the liver, the same factor in combination with constant illumination will lead to the development of hepatitis; in some animals, there were already signs characteristic of liver cirrhosis.

The mechanisms underlying the development and progression of liver pathologies are not completely clear [47], but there are a number of common features in the pathogenesis of ALD and NAFLD [48]. The basis of alcoholic liver disease, as well as that of NAFLD, which can be combined, is steatosis (fatty liver), which is considered a benign and reversible condition. The mechanism of transformation of steatosis into steatohepatitis includes several pathogenetic links that are identical in both non-alcoholic and alcoholic liver damage. In the case of persistence of the damaging factor (in NAFLD, the state of insulin resistance), regeneration slows down and hepatocytes are replaced by an excess amount of extracellular matrix proteins, including fibrillar collagen—the distribution of which depends on the damaging factor. NAFLD is characterized by a perihepatocellular centrilobular fibrosis, while for ALD, a pericentral or perisinusoidal fibrosis is characteristic. Fibrosis can progress to septa and cirrhosis formation. In addition, it has been shown that in the development of NAFLD, a disruption of the day/night cycle is one of the contributory factors [49].

The conducted study established that chronic alcohol intoxication under conditions of a fixed light regime over three weeks does not provoke considerable changes in the studied micromorphometric parameters of hepatocytes, which is associated with the short-term influence of ethanol on the liver.

In the liver of rats in the second experimental group, there were significant deviations in several parameters from the values of the control group (hypertrophy of hepatocytes, changes in diameters of nuclei, etc.).

According to some sources, hypertrophy of hepatocytes is observed in cases of polyploidization of nuclei and in the formation of binuclear cells; however, a decrease in the proportion of binuclear hepatocytes occurs in the liver of rats in this group, which indicates a suppression of proliferative processes [50]. It has been shown [51] that melatonin has the ability to activate the proliferation of hepatocytes by inhibiting *IKKα*, *JNK1* and *cJUN* (c-Jun N-terminal kinases), which oppress mitotic and apoptotic activity, and that the absence of pineal melatonin causes a decrease in mitotic activity [52]. It is known that increases in the number of binuclear hepatocytes is one of the effects of melatonin [53].

At the same time, a number of researchers have suggested that some functions of di- and polyploid hepatocytes may differ. It has been found that diploid hepatocytes accelerate liver regeneration caused by resection and can accelerate compensatory regeneration after acute injury, and that polyploid cells protect the organ from tumor initiation in hepatocellular carcinoma and promote adaptation to chronic damage [54,55,56]. Kreutzer C. [57] has proposed the consideration of nuclear ploidy as a new factor in hepatocyte diversity and has described various biological functions in polyploid and diploid hepatocytes.

In turn, an increase in the linear dimensions of hepatocytes after three weeks under constant illumination is the result of an increase in their functional activity. Growth in the size of hepatocytes can be an indicator of the activation of intracellular plastic processes that increase the energy capabilities of cells. Such changes have been described for the liver under conditions of chronic stress, such as the 21 day darkness deprivation.

Enhancements in the areas of hepatocytes are associated with an increase in levels of stress hormones. Stimulation of α1-adrenoreceptors increases the nuclear volume of hepatocytes and the density of nucleoli due to the release of intracellular Ca^2+^ and the subsequent activation of DNA polymerase. In addition, endotoxins and *TNFα* increase the volume of hepatocytes by activation of signaling pathways and retention of sodium and water [58,59].

In turn, the development of small-drop fatty degeneration in hepatocytes under stress is a described phenomenon; it correlates with the duration of stress exposure [60,61], is associated with an increase in the level of adrenal cortex hormones—primarily glucocorticoids, which cause an increase in the expression of serotonin *5-HT2A* and *5-HT2B* receptors and tryptophan hydroxylase 1—as well as in the synthesis of serotonin [62]. The accumulation of lipid droplets by hepatocytes under stress is accompanied by an increase in lipolysis gene expression and β-oxidation of fatty acids [63,64].

Increases in mean nuclear diameter, along with a simultaneous decrease in their elongation index and a tendency to increase in size, indicates the development of pathological changes in the nuclei [65].

In hepatocytes of rats of the third experimental group, the deviations in the values of the studied karyometric parameters (a decrease in the cross-sectional area, perimeter and volume of the nuclei, a decrease in the NCR as well as in the ratio of the volume of the nucleus to its area, and an increase in the proportion of binuclear hepatocytes) from the control parameters were more pronounced. At the same time, a decrease in the contour index against the background of an increased coefficient of form indicated ongoing processes of nuclear decomposition, which were more pronounced than in the previous groups—making it possible to identify them by previously described methods of light microscopy [66].

In the hepatocytes of the animals in this group, a significant increase in the expression of *Ki-67* was noted. These data testify that the combined effect of two stress factors leads to an intensification of the proliferative process, i.e., reparative regeneration.

In turn, an increase in *p53* expression in the hepatocytes of rats of all the experimental groups indicated an increase in the apoptotic activity of hepatocytes under the influence of the studied factors [67]. Decreases in the expression of *Bmal1* and increases in the expression of its antagonist *Per2* served as a confirmation of the chronodestructive effects of both constant lighting and ethanol. It is natural that the expression of *Adh5* was higher in the hepatocytes of rats that consumed ethanol.

As a result of the study, it was found that both alcohol intoxication and constant illumination cause significant changes in the structure of the CRs of the studied parameters, both as separate and combined actions. The CR of the area of the nucleus, although it changes under the influence of ethanol and constant illumination, acting separately, is disrupted only under the combined action of these two factors.

The most stable and least labile CR was the CR of the cell area. Being disrupted in the liver of rats of the first experimental group, it was present in the second and third groups, with characteristics close to the control group. The influence of alcohol manifested itself differently in relation to this parameter, depending on the lighting regime—causing rhythm disruption under a fixed light regime, but not exerting the same effect under constant lighting.

We did not find an explanation for this fact in the available literature. However, it can be assumed that in the first experimental group, ethanol, influencing the pineal gland, causes a disturbance in the rhythm of the functioning of this organ—indirectly causing the destruction of the CR of the cell area. In animals of the second and third experimental groups, under constant illumination, there was no production of pineal melatonin, but it can be assumed that the role of pacemaker in this case was played by some other structure or process that does not depend on the action of ethanol and melatonin. This assumption is supported by the closeness of the CR acrophases in these groups. Intracellular processes associated with the regulation of the rhythms of the molecules that form the cytoskeleton of the cell can be proposed as candidates for this role. Thus, the maximum content of actin fibers in hepatocytes, and, accordingly, their largest sizes, was observed at 10:00 [68], and the acrophases of the rhythms of hepatocyte sizes established by us were at 10:13 in the control, 11:37 in the second and 10:09 in the third experimental groups.

It is noteworthy that the CRs of *p53* and *Ki-67* were observed only in the control group and in animals of the first group, albeit with an altered rhythm. Thus, the leading role in the destruction of these CRs belonged to the constant lighting condition. At the same time, the CRs of the clock genes were found only in the control and the second experimental group, which is caused by the leading role of ethanol in the disruption of these rhythms.

The rhythm of *Adh5* was disrupted only in the second group. Apparently, violation of the lighting regime causes its breakdown, and the presence of CRs in rats of the first and third experimental groups was caused by the fact that light was not the pacemaker, but the time at which the ethanol was drunk was.

Chronic alcohol intoxication causes a complex of metabolic disorders that complicate the toxic effects of alcohol. In turn, dark deprivation is a significant stress factor that also affects metabolism.

Thus, the hyperglycemia we found in animals of the first and second experimental groups is explained in the first case by the well-known effect of CAI, which can cause disorders of carbohydrate metabolism, manifesting in both hyper- (more often) and in hypoglycemia [68,69], and in the second case by a response to stressful effects. At the same time, a significant decrease in the levels of this metabolite in the blood plasma of rats of the third group was caused by a disruption of the adaptation process. In addition, hyperglycemia in the blood of rats of the first group could be explained by the development of gluconeogenesis, since overexposure to ethanol is known to lead to insulin resistance in the liver, which increases the enzymatic capacity of gluconeogenesis and lipogenesis and decreases glycogen synthesis by inhibiting the PI3K/AKT signaling pathway in the liver [70].

The level of transaminases in the blood plasma of rats of the first group did not differ from the controls; in the second group we noted an increase in the levels of both ALT and AST, but in the third group only the level of AST was increased. Changes in the levels of enzymes in the second group may be associated with the destruction of hepatocytes under constant illumination, and the picture in the third group is explained by the combined effect of both factors; an isolated increase in AST may be associated with a pyridoxine (vitamin B_6_) deficiency that often develops during CAI, as a result of which, ALT activity in hepatocytes decreases. In addition, alcohol promotes the release of mitochondrial AST from hepatocytes without their obvious damage. A decrease in the level of total protein was noted in the blood plasma of rats of all the experimental groups. Hypoproteinemia is a well-known phenomenon that disrupts the morphofunctional integrity of the liver. It is noteworthy that a decrease in albumin levels occurred only in rats of the second experimental group, which suggests that constant illumination causes significant disturbances in the protein-synthesizing function of the liver [71,72].

An increase in the level of direct bilirubin in the blood of rats of the first and third experimental groups is a well-known effect of CAI.

The rhythms of glucose metabolism are determined by diurnal variations in a variety of metabolic pathways, including peripheral insulin sensitivity, β-cell sensitivity, insulin clearance, the sleep–wake cycle, etc. [73]. The existence of various circadian phenotypes in humans has also been shown [74], which suggests their presence in rodents. The CRs of glucose appears to be the most stable, being destroyed in none of the groups, although they did undergo some changes.

We found that the circadian rhythm of ALT was more resistant to the effects of the investigated factors, being disturbed only in rats of third group, while the CRs of AST were absent in rats of all the experimental groups.

It is notable that both the CRs of total protein in the blood plasma and of albumin, being altered in animals of the first and second experimental groups, were destroyed in the third group; this testifies, on the one hand, to the resistance of these rhythms to the effects of external desynchronizers, and also to the strong chronodestructive effects of the combination of ethanol and constant illumination.

The least resistant CRs to the action of the investigated chronodestructors were the CRs of total and direct bilirubin, which were destroyed in all the experimental groups.

## 4. Materials and Methods

### 4.1. Object of the Study

This study was conducted on 160 male rats of Wistar outbred stock at an age of 6 months, with a body weight of 350 g. Animals were taken from the “Stolbovaya” affiliate of the FSBIS Scientific Center for Biomedical Technologies of the Federal Medical and Biological Agency. All animals were kept for three weeks in standard laboratory conditions, in plastic cages with free access to water and food. Initially, the animals were kept in natural lighting, at a temperature of 20–22 °C and a relative humidity of 60–70%. The rats had free access to drinking water and briquetted food. Keeping of animals and experiments were performed in accordance with the European Convention for the Protection of Vertebrate Animals used for Experimental and other Scientific Purposes (Strasbourg, 18 March 1986). This research was approved by the Bioethical Committee of the Federal State Budgetary Scientific Institution “Research Institute of Human Morphology”, protocol № 27/3 dated 11.10.2021.

### 4.2. Design of Study

Rats were divided into 4 equal groups. To model the KhAI we used a 15% aqueous solution of ethanol [75].

The control group (*n* = 40) was kept under a fixed light regime (light:dark/10:14 h with lights on at 8:00 and off at 18:00).

The first group, (EtOH; *n* = 40) was kept under the same conditions as the controls, but a 15% aqueous ethanol solution was offered daily as a drink *ad libitum* instead of water.

The second group, (CL; *n* = 40) was kept under a regime of constant light.

The third group (EtOH + CL; *n* = 40), was kept under a regime of constant light and received as a drink a 15% aqueous solution of ethanol *ad libitum*.

The criterion for selecting rats for the study, along with the absence of visible abnormalities in condition and behavior, was the initial preference for a 15% solution of ethyl alcohol in comparison with tap water. For this, a preliminary experiment was carried out for 3 days in individual cages with free access to both liquids.

During the experiment, the volume of the consumed ethanol solution was determined daily, and then the mass of alcohol per 1 kg of body weight was calculated. On average, the animals drank 15.48 ± 1.28 mL/day, which in terms of absolute ethanol is 7 g/kg of body weight.

Euthanasia was carried out three weeks after the start of the experiment in a carbon dioxide chamber equipped with a device for the upper gas supply (100% CO_2_) at 9.00, 15.00, 21.00 and 3.00. The chamber volume was filled with gas at a rate of 20% per minute to avoid dyspnea and pain in animals. Previously, the rectal temperature of the animals was measured and blood sampling for hematological and biochemical studies was made. After sacrifice, evisceration was performed.

### 4.3. Morphological, Morphometric and Histochemical Methods

The liver was fixed in 10% neutral buffered formalin with further passage through alcohols of increasing concentration (50°, 60°, 70°, 80° and 96°) and xylol, followed by pouring into Histomix histological medium (BioVitrum, Moscow, Russia). When conducting studies of organs embedded in paraffin, serial sections with a thickness of 5–6 μm were prepared. Histological sections were made on the rotor microtome MPS-2 (USSR). Hematoxylin–eosin staining was carried out according to the standard technique. Stained sections were put into a BioMount mounting medium (BioVitrum, Moscow, Russia).

Fragments of the liver were frozen for subsequent histological examination, and using a freezing table for the MFT -01 “Unicon” microtome, serial frozen sections with a thickness of 6–8 μm were prepared. To confirm the presence of fatty degeneration, standard staining of frozen sections with a solution of Sudan-III in 70% ethyl alcohol was performed.

Microscopy of histological preparations was performed using a Nikon Eclipse 80I microscope with use of a Nikon DI-FI digital camera (Tokyo, Japan). Eyepieces ×10, ×15, objectives ×4, ×10, ×20, ×40, ×100 were used for microscopy. Microscopy of histological preparations was performed using a Nikon Eclipse 80I microscope with the use of a Nikon DI-FI digital camera (Tokyo, Japan). From each studied preparation, 10 digital images of randomly selected visual fields were taken at a magnification of ×400, ×1000, with the use of which karyo- and cytometry were subsequently carried out. In morphometric studies, the Fiji software package, a program built on the basis of ImageJ v2 with appropriate plugins, was used [76]. The measurements were carried out in micrometers after preliminary geometric calibration on an object-micrometer scale digitized with the same magnification. Micromorphometry was performed only for mononuclear interphase hepatocytes without signs of pathological changes. The proportion of binuclear hepatocytes was also determined.

With the use of ImageJ, the cross-sectional areas of nuclei (area of nucleus, S_n_), the small (d) and long (D) diameters of nuclei, the perimeters of nuclei (P_n_), the cross-sectional areas of cells (area of cell, S_cell_), and the small (a) and long (b) diameters of cells were determined.

The parameters were calculated with the use of appropriate formulae.

The nuclear-cytoplasmic ratio was calculated by the formula:

NCR = S_n_/S_c_ (1)

where: S_n_—area of nucleus of cell; S_c_—area of cytoplasm.

The mean diameters of the nuclei were calculated by the formula:

M = (D + d)/2(2)

where D—long diameter, d—small diameter [77].

The volumes of the nuclei were calculated by the formula:

V_n_ = 0.523 M^3^ (3)

where M—mean diameter of nuclei.

The volumes of the cells were calculated by the formula:

V_c_ = 0.523 M^3^ (4)

where M—mean diameter of cells.

The nucleus volume to nucleus area ratio (V/A coefficient) was calculated by the formula:

V_n_/A_n_ (5)

where V_n_ is the mean volume of nuclei, A_n_—mean area of nuclei.

The elongation index of the nucleus was calculated by the formula:

EI = D/d(6)

where D—long diameter, d—small diameter [76].

For calculation of the coefficient of form, the following formula was used:

CF = 4 × π × S_n_/P_n_^2^ (7)

where S_n_—area of nucleus, P_n_—perimeter of nucleus [48].

The contour index of the nucleus, which reflects the topography of its surface, was determined by the formula:

CI = P_n_/√ S_n_ (8)

where S_n_—the area of nucleus, P_n_—perimeter of nucleus [77].

### 4.4. Immunohistochemical Methods

To carry out immunohistochemical reactions, liver sections were dewaxed, rehydrated and treated with 3% hydrogen peroxide solution to block endogenous peroxidase. Then, the slices were put into an Ultra V Block (Thermo Fisher Scientific, Waltham, MA, USA) solution; the antigens were previously unmasked by boiling in citrate buffer (pH 6.0). Immunohistochemical reactions with primary antibodies were performed.

The following antibodies were used:

*Ki-67*—Rabbit polyclonal (Cloud-Clone Corp., Katy, TX, USA), 1:300;
*Per2*—Rabbit polyclonal (Cloud-Clone Corp., Katy, TX, USA), 1:200;
*Bmal1*—Rabbit polyclonal (Cloud-Clone Corp., Katy, TX, USA), 1:200;
*p53*—Rabbit polyclonal (Cloud-Clone Corp., Katy, TX, USA), 1:200;
*Adh5*—Rabbit polyclonal (Cloud-Clone Corp., Katy, TX, USA), 1:300.

Sections were incubated with antibodies for 60 min at room temperature. The UltraVision Quanto Detection System (Thermo Fisher Scientific; Waltham, MA, USA) set was used as a detection system.

Reactions with replacement of primary antibodies with phosphate buffer solution served as controls.

After the sections acquired a blue hue, the slides were removed, dehydrated in alcohols of ascending concentrations and xylene according to the standard scheme, and embedded in the BioMount mounting medium (BioVitrum, Moscow, Russia).

The results of the immunohistochemical reactions were assessed by the proportion of stained cells or cell nuclei (depending on the localization of the antigen) in relation to the total number of hepatocytes. The evaluation was carried out in 4 fields of view at a magnification of × 400.

Cells stained with the appropriate antibodies were counted in the preparations, and then the corresponding index was calculated as the ratio of stained cells to the total number of cells (%).

### 4.5. Biochemical Methods

The levels of the studied parameters were determined in blood plasma using a StatFax-3300 analyzer (Palm City, FL, USA) with the corresponding Spinreact kits (Barcelona, Spain): total protein, albumin, alanine aminotransferase (ALT), aspartate aminotransferase (AST), direct bilirubin, total bilirubin, and glucose were determined.

### 4.6. Methods for Statistical Processing

The obtained data were analyzed using the “GraphPad Prism 6.0” software by calculating the mean values, standard deviation, and mean error of the arithmetic mean. The data in the text, tables and graphs are presented as M ± m, where M is the mean and m is the SD. Numerical rows characterizing the daily fluctuations of the studied physiological rhythms of animals were subjected to mathematical processing, on the basis of which the group chronograms were drawn. The shapes of the chronograms were studied and the average daily values were calculated. To identify differences between control and experimental groups, we performed univariate analyses (using ANOVA or Kruskal–Wallis where appropriate) for clinical parameters and laboratories. Differences were considered statistically significant at *p* < 0.05.

For statistical calculation of the amplitude and acrophase of CRs, we performed cosinor analysis, which is an international, generally recognized method for the unified study of biological rhythms, using the CosinorEllipse2006-1.1 program.

Cosinor analysis was employed to analyze wave processes and process chronobiological data. The presence of a reliable circadian rhythm was determined, as well as its acrophase and amplitude. The output information of the cosinor analysis was the main parameters of the rhythms: mezor, i.e., the value of the average level of the sinusoid (h); the amplitude of the sinusoid (A); and the acrophase (Phi), that is, the time of the onset of the maximum of the function. The mezor coincides in magnitude with the average daily value of the investigated function. The acrophase is a measure of the peak time of total rhythmic variability over a 24 h period, i.e., the time of the onset of the maximum of the function. The amplitude corresponds to half of the total rhythmic variability in the cycle. The acrophase is expressed in hours; amplitude values are expressed in the same units as the studied variables [78].

## 5. Conclusions

The results of the micromorphometry indicated significant changes in the studied parameters of the hepatocytes, which were least expressed in the first experimental group, and which affected the liver cells of the rats of the third experimental group the most. In the hepatocytes of animals of all three experimental groups, there was a significant change in the chronostructure, which manifested itself in the disruption and/or alteration of the studied CRs and the occurrence of desynchronosis. The most stable and least labile CR was the CR of the cell area. In the presence of changes in the biochemical parameters typical of ethanol intoxication, a significant change or destruction of their CRs in all the studied groups of animals was noted. Both ethanol and constant illumination caused changes in the level of expression and circadian rhythms of the studied genes; however, the greatest chronodestructive effect was observed with the combined actions of these factors.

Alcohol intoxication, which occurred against a background of a melatonin deficiency caused by constant lighting, was accompanied by a significantly greater severity, prevalence, and intensity of inflammatory tissue reactions and a variety of manifestations of liver pathology in all animals. Under these conditions, understanding the mechanisms of the modeled pathology opens up prospects for both etiotropic treatment and pathogenetic targeting.

## Figures and Tables

**Table 1:** Results of the micromorphometric study of hepatocytes of rats.

	Control	EtOH	CL	EtOH + CL
**Cross-sectional area of nucleus, μm^2^**	41.79 ± 8.13	42.65 ± 4.80	42.72 ± 5.63	35.50 ± 3.01 ***
**Volume of nuclei, μm^3^**	205.90 ± 59.54	210.51 ± 35.39	211.30 ± 41.67	159.59 ± 20.23 **
**Nucleus volume to nucleus area ratio (V/A coefficient)**	4.84 ± 0.47	4.90 ± 0.27	4.90 ± 0.32	4.48 ± 0.19 ***
**Perimeter of nuclei, μm**	14.96 ± 4.78	15.34 ± 4.78	15.11 ± 3.39	9.59 ± 2.49
**Mean diameter of nuclei, μm**	7.25 ± 0.91	7.34 ± 0.89	7.77 ± 0.75 **	7.17 ± 0.76
**Elongation index of nuclei**	1.23 ± 0.06	1.22 ± 0.08	1.14 ± 0.05 ***	1.19 ± 0.07
**Coefficient of form**	2.35 ± 0.11	2.28 ± 0.15	2.35 ± 0.11	4.85 ± 0.17 *
**Contour index**	2.31 ± 0.10	2.35 ± 0.13	2.31 ± 0.11	1.61 ± 0.18 ***
**Cross-sectional area of cell, μm^2^**	185.80 ± 31.95	190.10 ± 34.03	261.90 ± 55.30 ***	184.80 ± 21.67
**Volume of cell, μm^3^**	1926.01 ± 486.21	1994.02 ± 510.02	3317.21 ± 818.81 ***	1898.25 ± 326.85
**NCR**	0.230 ± 0.056	0.233 ± 0.055	0.162 ± 0.022 ***	0.194 ± 0.018 **
**Proportion of binuclear hepatocytes, %**	7.44 ± 2.66	8.92 ± 3.60	4.73 ± 2.03 **	6.51 ± 2.56

Note: hereafter, * *p* ≤ 0.05; ** *p* ≤ 0.005; *** *p* ≤ 0.0005—statistical significance of differences in comparison with the control group.

**Table 2:** Cosinor analysis of the micromorphometric parameters of rat livers.

Area of Nuclei of Hepatocytes, μm^2^		
	Amplitude	Acrophase
**Control**	10.03	12^21^
**EtOH**	3.73	18^02^
**CL**	4.60	11^36^
**EtOH + CL**	No reliable CR	
**Area of Hepatocytes, μm^2^**		
**Control**	24.84	10^13^
**EtOH**	No reliable CR	
**CL**	40.02	11^37^
**EtOH + CL**	16.84	10^09^
**NCR**		
**Control**	0.033	14^01^
**EtOH**	No reliable CR	
**CL**	No reliable CR	
**EtOH + CL**	0.017	19^46^

**Table 3:** Cosinor analysis of the biochemical parameters of rat livers.

	Amplitude	Acrophase
Glucose, mmol/L		
**Control**	13^08^	1.50
**EtOH**	14^32^	2.06
**CL**	12^06^	0.70
**EtOH + CL**	10^01^	0.45
**ALT, U/L**		
**Control**	2^08^	0.80
**EtOH**	No reliable CR	
**CL**	No reliable CR	
**EtOH + CL**	No reliable CR	
**AST, U/L**		
**Control**	15^15^	13.80
**EtOH**	11^15^	26.90
**CL**	22^31^	17.15
**EtOH + CL**	No reliable CR	
**Total Protein, g/L**		
**Control**	14^48^	6.14
**EtOH**	18^41^	8.70
**CL**	10^46^	8.06
**EtOH + CL**	No reliable CR	
**Albumin, g/L**		
**Control**	14^16^	9.57
**EtOH**	13^19^	3.47
**CL**	3^24^	9.64
**EtOH + CL**	No reliable CR	
**Direct Bilirubin, μmol/L**		
**Control**	1^48^	3.48
**EtOH**	No reliable CR	
**CL**	No reliable CR	
**EtOH + CL**	No reliable CR	
**Total Bilirubin, μmol/L**		
**Control**	23^35^	8.10
**EtOH**	No reliable CR	
**CL**	No reliable CR	
**EtOH + CL**	No reliable CR	

**Table 4:** Results of the immunohistochemical study on rat hepatocytes, * *p* < 0.05, ** *p* < 0.01, *** *p* < 0.001.

	Control	EtOH	CL	EtOH + CL
**Ki67, %**	1.0 ± 0.17	1.03 ± 0.5	1.35 ± 0.56	5.73 ± 0.35 ***
**p53, %**	2.2 ± 0.11	4.73 ± 0.14 ***	3.99 ± 0.13 **	3.20 ± 0.12 **
**Bmal1, %**	60.76 ± 2.04	41.05 ± 2.06 ***	16.40 ± 1.32 ***	22.61 ± 1.15 ***
**Per2, %**	31.13 ± 1.68	46.37 ± 1.87 **	40.01 ± 3.30 **	39.60 ± 0.70 **
**Adh5, %**	5.28 ± 0.36	23.18 ± 1.30 ***	5.11 ± 0.4	33.55 ± 1.34 ***

**Table 5:** Cosinor analysis of biochemical parameters in the livers of rats.

	Amplitude	Acrophase
Ki-67, %		
**Control**	22^14^	1
**EtOH**	10^24^	1.01
**CL**	No reliable CR	
**EtOH + CL**	No reliable CR	
**p53, %**		
**Control**	20^48^	0.35
**EtOH**	13^42^	0.6
**CL**	No reliable CR	
**EtOH + CL**	No reliable CR	
**Bmal1, %**		
**Control**	13^34^	6.28
**EtOH**	No reliable CR	
**CL**	9^48^	6.7
**EtOH + CL**	No reliable CR	
**Per2,%**		
**Control**	4^02^	8.04
**EtOH**	No reliable CR	
**CL**	13^04^	14.06
**EtOH + CL**	No reliable CR	
**Adh5, %**		
**Control**	9^13^	1.19
**EtOH**	3^24^	9.5
**CL**	No reliable CR	
**EtOH + CL**	2^14^	8.68

## Data Availability

The data presented in this study are available within the article text, tables and figures.
